# Testing the influence of testosterone administration on men’s honesty in a large laboratory experiment

**DOI:** 10.1038/s41598-018-29928-z

**Published:** 2018-08-01

**Authors:** Austin Henderson, Garrett Thoelen, Amos Nadler, Jorge Barraza, Gideon Nave

**Affiliations:** 1 University of California San Diego, Rady School of Management, San Diego, 92093 United States; 2 Claremont Graduate University, Department of Politics and Economics, Claremont, 91711 United States; 3 Western University, Ivey Business School, London, ON N6G 0N1 Canada; 4 University of Southern California, Dornsife, Los Angeles, 90089 United States; 5 University of Pennsylvania, Wharton School of Business, Philadelphia, 19104 United States

## Abstract

The impact of testosterone on decision-making is a growing literature, with several reports of economically relevant outcomes. Similar to Wibral *et al*. (2012), we investigate the effects of exogenous testosterone administration on deception in a double-blind placebo controlled study. Participants (*N* = 242) were asked to roll a die in private and were paid according to their reported roll, which creates the opportunity to lie about the outcome to increase earnings. We find evidence for self-serving lying in both treatment and control groups and a statistically insignificant negative effect (*d* = −0.17, 95% CI[−0.42, 0.08]) indicating more honest behavior (i.e., lower reports) following testosterone administration. Although insignificant, the direction was the same as in the Wibral *et al*. study, and the meta-analytic effect of the two studies demonstrates lower reporting (i.e., more honesty) following testosterone (vs. placebo) administration, significant at the 0.05 level (*d* = −0.27, 95% CI[−0.49, −0.06]). We discuss how our results and methodology compare with Wibral *et al*. and identify potential causes for differences in findings. Finally, we consider several plausible connections between testosterone and lying that may be further investigated using alternative methodologies.

## Introduction

Lying plays an important role in interpersonal relationships and many types of economic transactions, as it can create strategic advantages from informational asymmetries. Investigations of the determinants of lying have recently attracted widespread attention, and include research of the roles played by other-regarding preferences^1^, social and cultural norms^2,3^, the size and nature of incentives^4–6^, the likelihood and costs of detection^7^, performance in an antecedent competition^8^, the opportunity for self-justification or self-signalling^9–11^, and the role of individual differences, and gender in particular^12,13^.

Deception is a part of the behavioral repertoire of many animal species^14–16^. The understanding of the biological foundations of deceptive behavior or lying in humans, however, is limited. Functional Magnetic Resonance Imaging (fMRI) studies suggest that deception is associated with increased activation in brain regions involved in socio-cognitive processes, such as the right tempero-parietal junction, precuneus and anterior frontal gyrus, and executive functions, such as the anterior cingulate cortex and amygdala^17–20^. In addition, two studies reported that intranasal administration of the neuropeptide oxytocin promotes group-serving dishonesty^21^ and decreases the ability to detect lies told by members of the opposite sex^22^. However, it should be noted that several methodological reviews have recently challenged the validity of the intranasal oxytocin literature, casting uncertainty over these findings^23–25^.

The male sex steroid hormone testosterone plays a central role in physical development, and has been shown to have considerable psychological effects, such as on mood in hypogonadal men^26,27^ and cognition^28–31^. There are also several reports documenting the hormone’s impact on decision making in a variety of economically important contexts, such as financial risk taking^32^, asset trading^33–35^, and economic games assessing trust, reciprocity, and cooperation^36–38^.

While much of testosterone behavioral research has focused on antisocial behaviors, such as aggression^39,40^, testosterone has also been shown to promote prosocial behavior in certain contexts, such as increasing fair bargaining behavior^38^. A common explanation for testosterone’s promotion of prosocial behavior in some contexts and antisocial behavior in others is that testosterone may increase the desire for social status and thus promotes status seeking behavior^41–43^. Along this line of argumentation, lying is a socially complex behavior that can affect social status. Hence, testosterone may impact lying in ways that increase social status, even at an economic cost. Consistent with this notion, a study of Dutch females who were administered testosterone before playing bluff poker found that the participants who received testosterone were less likely to bluff and more likely to call bluffs^44^. The authors argued that while random bluffing was the payoff maximizing strategy in the game, exhibiting dishonesty was harmful for the player’s social status.

In a study closely related to our own, Wibral *et al*.^45^ investigated the influence of testosterone administration on lying with a die-roll task (originally introduced in Fischbacher & Heusi^4^) — an active behavioral measure of deception that has also been shown to predict dishonest behavior in the field^46^. In this study, German male participants (*N* = 91) were randomly administered testosterone or placebo under a double-blind exogenous administration protocol and were given monetary incentive to lie without possibility of being discovered. Wibral *et al*. found that testosterone, in comparison to placebo, significantly reduced deception. The authors speculated that this decrease in lying was caused by testosterone’s effects on pride and self-image, two psychological constructs that are related to status concerns but do not require the actualization of status outcomes to impact behavior.

The current study aims to further test the robustness and generalizability of the findings of Wibral *et al*., because of the following reasons. First and foremost, recent large scale investigations have repeatedly demonstrated the importance of building a robust epistemological foundation that allows science to progress cumulatively^47^. While an encouraging fraction of laboratory economics experiments can be successfully replicated, a considerable proportion of significant effects either cannot be replicated or the replicated effect size is of a smaller magnitude^48^.

It should be noted that this experiment is not a direct replication of the Wibral *et al*. study. Although the overall experimental design is similar, several methodological modifications (detailed later in Differences from Wibral *et al*.^45^) were purposefully made to increase our likelihood of detecting behavioral effects from testosterone administration. Such iterative methodological changes, along with employing ample sample sizes, are important for testing for the robustness and generalizability of the effect.

Second, although the effect reported by Wibral *et al*. is seemingly strong and with a relatively small *p*-value (2-sided t-test, *t*(89) = 2.65, *p* < 0.001), the die-roll task was a part of an experimental battery comprising of 11 tasks - a common research practice in behavioral endocrinological research that is aimed at maximizing the knowledge gained from each participant undergoing a pharmacological treatment^49^. The Bonferroni corrected *p*-value is *p* = 0.11, which means that the statistical evidence were not overwhelming.

Finally, while the sample size (*N* = 91) was larger than previous testosterone administration studies, the number of participants who faced an opportunity to lie was effectively smaller, due to the random nature of the task. This is because participants whose die roll outcome is high face no incentive to misreport.

To this end, we conducted a double-blind placebo-controlled investigation of exogenous testosterone’s effect on the die-roll task, in a sample of *N* = 242 American participants (mostly college students, for full demographic details see Supplementary Materials S1). Our sample size is over 2.5 times larger than the Wibral *et al*.^45^ study - a magnitudinal difference that is in line with the “small telescope” heuristic which provides the statistical power to test whether the original study was underpowered to detect the reported effect size^50^. As in the Wibral *et al*. study, participants were seated privately in cubicles without the possibility of observation by researchers or other participants. They were each given a 6-sided die, a pen, and a slip of paper, and instructed via computer to roll the die and report the outcome both on the slip of paper and into the computer, thus earning them the dollar amount of what they reported. As in the original study, the task was a part of an experimental battery. Given the Wibral *et al*. report, we hypothesized that participants who received testosterone would be more honest (i.e., report lower outcomes compared to placebo).

## Methods

### Participants

Males over the age of 18 (mean = 23.65, SD = 7.24), mostly college students, were recruited via e-mail and posters to participate in an experiment on testosterone and economic decision making at the Center for Neuroeconomics Studies, Claremont Graduate University. 125 participants were administered testosterone gel and 118 were administered a placebo gel. One participant who was administered the placebo gel left the experiment before participating in the die-roll task and was therefore excluded from all analyses, bringing the total number of participants used in analysis to *N* = 242. The institutional review boards of Caltech and Claremont Graduate University approved this study, all participants gave informed consent, and no adverse events occurred. The study was performed in accordance with the guidelines set forth by both IRBs. Descriptive statistics of the participants are presented in Supplementary Materials Table S1.

### Procedure

In each experimental day there were two sessions, with one in the morning and one in the afternoon. The morning session lasted from 9:00 am to 9:45 am, and the afternoon session lasted from 2:00 pm until roughly 4:15 pm. Participants provided 4 saliva samples, one in the morning, and three in the afternoon. Participants completed the die roll task immediately before the 4th saliva sampling, which took place on average at 4:17 pm (SD = 12.2 minutes), and were dismissed shortly thereafter.

Chronologically, participants arrived at the laboratory in the morning in groups of 12 or 16, whereupon they were given an informed consent form and signed it upon assent. They then proceeded to a separate room where their hands were scanned (digit ratio is a purported measure of pre-natal testosterone exposure^51^) and facial photographs were taken (facial characteristics are associated with testosterone levels^52^). Next, they went to another room where they completed brief demographic and mood surveys in randomly assigned private cubicles. The private cubicles had a desk, computer, keyboard, monitor, and mouse, and all activity on the computer or desk was out of sight of any other participant or researcher. A saliva sample was taken at the cubicles to assess baseline testosterone levels, the first of a total of 4 samples taken for each participant (the three others were taken during the afternoon session).

Participants then proceeded to another separate room in groups of 2–6 where they were given a small paper cup containing either 10 g of topical testosterone 1% (2 × 50 mg packets Vogelxo^®^ by Upsher-Smith) or volume equivalent of an inert placebo of similar texture and viscosity (80% alcogel, 20% Versagel^®^) under a double-blind protocol (the paper cups were filled by the lab manager, who did not interact with the participants or reveals its contents to the research assistants). Participants were instructed to remove their shirts and self-apply the entirety of the cup’s contents to their shoulders, upper arms, and chest, as demonstrated by a research assistant. Participants were also instructed to not put their shirts back on until the gel had fully dried. Following application of the gel, all participants were asked to avoid touching any part of their body before washing their hands, and then brought into an adjacent restroom in order to thoroughly wash their hands with warm water and soap.

Participants were then given a strict set of instructions (which were also in the informed consent and recruitment materials), both verbally to the group and on a printed hand-out given to each participant, of what to do preceding the afternoon session and for the next 23 hours. Participants were told to refrain from bathing or any activities that might cause excessive perspiration, not to eat after 1:00 pm (in order to produce high quality saliva samples), and to return to the lab by 1:55 pm. Participants were then dismissed from the laboratory until the afternoon session. They were also told to abstain from any skin-to-skin contact with females, as per the recommendations of the testosterone gel manufacturer. A researcher contacted each participant via text message shortly before 1 pm to remind them to not eat any more and that they were only allowed to consume water before the afternoon session. Upon return for the afternoon session, a researcher verbally confirmed with each participant whether they had adhered to the guidelines, and no participants admitted noncompliance. Participants were not allowed to drink water for the 10 minutes preceding a saliva sample, which was enforced via observation by researchers. Saliva samples were also inspected for abnormalities, e.g. whether it was dark from smoking or oral bleeding, and any such were marked in an experimental log for monitoring and potential exclusion.

For the afternoon session, participants returned to the same private cubicle they had used in the morning session. They then provided a second saliva sample at 2:05 pm. In each cubicle there were also a standard 6-sided die, slip of paper, and pen, which were used in the die-roll task. Upon arrivals (with no incidents of lateness) participants took part in a battery of seven behavioral tasks that included math-based competitions, risk preference questionnaires, the cognitive reflection test, and others as part of another experiment. The third saliva sample was taken at 3:15 pm, and the fourth sample was taken at 4:15 pm, with the die task immediately preceding the fourth sample. Once the researcher had collected the reported rolls from all participants, the participants were paid in cash for their earnings in the study, and then provided their final saliva samples.

### Die-roll task

Through Qualtrics, an online survey platform, participants were given instructions to roll the die on their desk, and both record the result of the die roll into the survey and onto the slip of paper (see Supplementary Materials S2 for full instructions). The instructions informed participants that they would receive the dollar value of their reported roll - a report of 3 would earn $3, a report of 5 would earn $5, *et cetera*. The instructions stated that participants could roll the die more than once, but that only their first roll would count. Once participants had recorded their roll, they brought their slips to the research assistant, who was standing at the far end of the room, on the other side of the cubicle walls that surrounded each participant. This ensured that the roll and recording of the roll outcome were both done privately.

### Differences from Wibral *et al*

In this section, we consider the *a priori* differences between our study design and that of Wibral *et al*.^45^, and how these differences may impact results. These differences are summarized in Table 1.

**Table 1:** Methodological differences between this study and Wibral *et al*.^45^.

	Wibral *et al*.^45^	This study
1. Testosterone Loading Time	21–24 hours	Approximately 7 hours
2. Administration method & Dosage	Topical gel application: 1 packet of Testogel^®^, which contains 5 grams of total gel and 50 mg testosterone (1% concentration).	Topical gel application: 2 packets of Vogelxo^®^ Gel 1%, each of which contains 5 grams of total gel and 50 mg testosterone (1% concentration), for a total of 100 mg of testosterone. The two packets were opened and pre-combined by the lab manager into a disposable cup before experimental sessions in order to preserve the double-blind for participants and researchers who interacted with participants.
3. System of Payoffs	Subjects receive Euro amount for reporting 1–5, but 0 for reporting a 6.	Subjects receive dollar amount for reporting 1-6.
4. Antecedent task (see SOM S6 for full list of tasks in battery for each experiment)	“Devil’s Task”, a risk preference task in which participants make a series of increasingly risky choices.	A risk task where participants were ranked by their performance.
5. Method of Measurement	Blood serum	Saliva
6. Subject Pool	German	American

First, our research differs from that of Wibral *et al*. in the loading period of testosterone. Our choice of testing schedule was based on the recommendations of a report by Eisenegger *et al*.^53^ which studies the pharmacokinetics of testosterone in healthy young men. The study documented clear elevation in testosterone levels between 3 and 7 hours after topical administration. The Eisenegger *et al*. report explicitly recommended testing for behavioral effects 7 hours after administration, and noted that peak testosterone levels were at 3 hours after administration. The findings of the Eisenegger *et al*. report are qualitatively similar to those of Chik *et al*.^54^, who also find that a transdermal application of testosterone (of lower dose than Eisenegger *et al*. or our study) in healthy young men led to peak serum testosterone levels roughly four hours after administration. In the Wibral *et al*. study, the die roll task took place about 21–24 hours after administration (thus between 18 and 21 hours after peak testosterone levels), whereas in our study it took place roughly 7 hours after testosterone administration, as suggested by Eisenegger *et al*.^53^.

One reason to be concerned that this methodological difference might cause the attenuation of the behavioral effect is due to lower treatment potency. Because our study used saliva sampling and Wibral *et al*. used blood sampling, we cannot directly compare measurements of testosterone levels. However, we confirmed a significant elevation in testosterone levels in our experiment, and this elevation did lead to behaviorally significant impacts in other tasks^31,43^. Relatedly, another study found that testosterone administration significantly increased aggression in some participants after only an hour^40^. Given the pharmacokinetics of testosterone, it is likely that this administration schedule led to higher testosterone levels in our study than in Wibral *et al*.^45^, and thus we would expect to see greater treatment potency (though we acknowledge that non-linear dose-dependency cannot be entirely ruled out). It should be noted, however, that the Eisenegger *et al*. study stopped sampling saliva after 7 hours, and more information is needed on the pharmacokinetics of testosterone over longer time periods as in Wibral *et al*.^45^.

Second, we differ in the amount of testosterone administered to participants. Whereas Wibral *et al*.^45^ used 50 mg, and the Eisenegger *et al*.^53^ study used 150 mg of topical testosterone gel, we decided to use 100 mg of testosterone gel. Our reasoning for using a larger dosage than Wibral *et al*. is that we wanted to increase the potency of our treatment in order to increase our probability of detecting the behavioral effects of testosterone. However, we did not increase the dose up to 150 mg, as in Eisenegger *et al*.^53^, in order to maintain ecological validity: 50 mg and 100 mg, but not 150 mg, are typical dosages indicated by prescription guidelines provided by the manufacturer of Vogelxo^®^. (the maximum recommended dose is 100 mg, with the advice to begin all patients at 50 mg daily for 14 days and adjust the dose upwards if serum testosterone levels are measured to still be below the normal range). The Eisenegger *et al*. report also notes that the pharmacokinetic data found in their study are qualitatively similar to those found by Chik *et al*.^54^, who studied the effects of 50 mg of testosterone in healthy young men. This suggests that the pharmacokinetics of the intermediate dose of 100 mg are likely to be similar as well.

Third, in the Wibral *et al*.^45^ study participants were paid the monetary value of their reported rolls of 1–5, but paid 0 for a reported roll of 6, where our study used a simpler payment scheme, with payoffs matching the reported roll. Although the salient decision individuals faced in either methodological design is essentially the same - whether to misreport a private die roll in order to increase earnings - this change does modify, to some degree, the stakes of the game - as in our study the worst a participant can do is earn $1, as opposed to nothing, and therefore so his incentive to lie may be reduced. However, as a meta-analysis of the die-roll task found no differences in reporting even when the differences in stakes are 500 times larger^55^, we find it unlikely that this difference made a substantial impact.

Fourth, in both studies the die-roll task was a part of an experimental battery (which is common practice in pharmacological experiments), but the batteries consisted of different behavioral tasks. In our experiment, the die roll was the last behavioral task, and it took place immediately following a task where participants made a series of either risky or safe bets, and then were publicly ranked and identified as “winners” and “losers” according to whether they were in the top or bottom half of earners. Previous research has shown that participants who win a competition tend to lie more afterwards in a die-roll game where the reported roll of one participant is subtracted from a shared amount to be split with another participant^8^. This study differs from our own in that reported rolls in our study did not impact the earnings of other participants. We test for any effect of winning or losing in the risk task, as well as an interaction with treatment, in our Results section. The antecedent task in Wibral *et al*.^45^ was the Devil’s Task, a risk preference measure in which participants either take or reject a series of gambles, wherein winning the gamble adds to a cumulative payoff or losing the gamble eliminates the entire payoff ^56^.

Fifth, in our study we used saliva to measure testosterone levels, compared to blood draws in Wibral *et al*. The advantage of using a saliva test is that it is operationally simpler as it does not require a blood draw. Relevant to the behavioral differences between the studies, blood draws cause some amount of pain and stress as compared to a saliva draw. This pain and stress could lead to an increase in cortisol levels, and the interaction between testosterone to cortisol might be important for deceptive behavior, as it is for aggressive behavior^57^. However, we did not find evidence that cortisol moderated the relationship between treatment and reported die roll in our study (OLS, coefficient for interaction between treatment and log cortisol levels *β* = 0.22, 95% CI[−0.43, 0.86], *p* = 0.51, see Supplementary Materials S6).

Last, participants in Wibral *et al*. were German and our study participants were American. This difference may have non-trivial consequences, as culture may influence perceptions of social status and actions which will lead to its elevation^58^. It may be the case that money is relatively more important for social status in America than Germany, and thus the same increased drive for social status will produce relatively less honesty among Americans. Propensities for honest behavior indeed vary substantially between cultures. For instance, one study found that in a task where participants were instructed to anonymously report the result of a coin flip (with a material incentive to misreport) only 3.4% of British participants lied, compared to 70% of Chinese participants^59^. While we are not aware of any studies that directly compare German and American behavior using similar methodologies, it is worth investigating if the impact of testosterone on behavior may differ according to cultural context in line with broader mechanisms associated with testosterone (e.g., status seeking).

### Measures

#### Saliva Sampling

A total of 4 saliva samples were taken throughout the experimental day, the 1st occurring before treatment administration between 9:25 and 9:34 am, the 2nd upon return to the lab for the afternoon session between 1:55 and 2:15 pm, the 3rd in the middle of the behavioral tasks battery between 3:02 and 3:38 pm, and the 4th at the very end between 4:10 and 4:44 pm. Participants were not allowed to bring food or drink into the laboratory, and the only water break allowed was immediately following the 3rd saliva sample, which occurred an hour before the 4th sample.

#### Hormonal Assays

Saliva samples were immediately stored on dry ice in coolers after collection and shipped to ZRT Laboratories (Beaverton, OR) for assay. Salivary steroids (estrone, estradiol, estriol, testosterone, androstenedione, DHEA, 5-alpha DHT, progesterone, 17OH-progesterone, 11-deoxycortisol, cortisol, cortisone, and corticosterone) were measured by liquid chromatography tandem mass spectrometry (LC-MS/MS) using an AB Sciex Triple Quad 5500. Further details about the assay procedure are available in the Supplementary Materials. A series of one-sample Kolmogorov-Smirnov tests for conformity to Gaussian (Supplementary Materials Table S2) indicated that all hormonal measurement distributions were better approximated by a Gaussian distribution following a log transformation, as indicated by higher p-values (i.e., the Gaussian normality hypotheses were less likely to be rejected after log-transformations). Thus, all hormonal measurements were log transformed prior to data analysis in order to make their distributions closer to Gaussian. It should be noted that these log transformations only impact our supplementary analysis, which is based primarily on OLS regression and is thus benefited from a normal distribution. Three saliva samples (two from sample 2, and one from sample 3) could not be analyzed due to insufficient fluid and thus excluded from analyses involving these hormonal samples.

After experimental session 13 (of 17) it was discovered that some of the pre-treatment baseline saliva samples from both treatment groups had testosterone measures exceeding those expected in healthy young men (i.e., greater than 400 pg/mL). Crucially, hormonal panel data show normal upstream and downstream testosterone metabolites dihydrotestosterone and androstenedione, respectively, among all participants with these abnormally high samples. Interpreting this singular hormonal abnormality, only the samples themselves were affected, but not participants’ physiological levels of testosterone. Following discovery of the viral spread into samples, a thorough experimental sterilization protocol was enacted, and the number of samples with abnormally high testosterone was drastically reduced. Ultimately, it was deduced that the testosterone gel had been transferred from common surfaces (e.g., door knobs, mouse pads) onto participants’ hands, and then into the saliva sampling tubes. Full details of the issue and our response are available in the Supporting Materials S4. In light of the resulting unreliability of measured testosterone levels, we avoid relying on measured hormones for analysis such as a regression of die rolls on testosterone levels; instead, we use treatment groups in our analysis.

#### Digit Ratio and Facial Masculinity

Digit ratio was calculated by first measuring the length of the second and fourth digits from the hand scans taken during the morning session, and then dividing the length of the second digit by that of the fourth. Facial masculinity was defined as the facial width-height ratio, calculated by measuring the distance between the cheekbones (width) and the upper eyelid to the top of the upper lip (height) and dividing width by height. Both facial and hand measurements were made using a software tool which counted the number of pixels between two points selected on an image. Two trained research assistants independently made each measurement, and the mean of the two measurements was used. Any discrepancies between the two measurements greater than 5% were reviewed by a senior researcher, of which there was only one instance. Further details on these measures are available in the Supplementary Materials.

### Statistical Approach

The first aim of our analysis is to provide a straightforward comparison between our results and those of Wibral *et al*. To that end, we perform the same set of statistical tests as those reported by Wibral *et al*., juxtapose their results against our own, and note differences. Our second aim is to make an assessment of the cumulative evidence on the relationship between exogenous testosterone and lying on the die roll task. To do so, we perform a joint analysis using a fixed effects model.

## Results

### Manipulation Check

We observed elevated levels of T and its metabolites (e.g., dihydrotestosterone) in the saliva measurements of the testosterone group but not in the placebo group following gel administration relative to baseline, and average levels of testosterone were significantly higher in the testosterone group than in the placebo group following gel administration. In order to verify that the participants who had received testosterone gel indeed experienced an elevation in their testosterone levels compared to those who received placebo, we submitted the logged testosterone levels to a repeated-measures ANOVA, that included treatment status as a between-subject factor, measurement time as a within subject factor, and the interaction between the two. The F-ratio of the interaction term was significant at the 0.01 level (*F*(3, 716) = 311.58, *p* < 0.001), indicating unequal mean levels of testosterone across sampling points and treatment status. We further tested for differences in logged testosterone levels between the two treatment groups in each of the four time point of saliva sampling, using 2-sided t-tests. Comparing log testosterone levels in the morning baseline sample across treatment groups yielded a non-significant difference (*t*(239) = 1.440, *p* = 0.15). The mean (SD) non-logged testosterone levels in the morning were 480.13 (826.95)pg/mL in the treatment group, and 616.24 (1052.93)pg/mL in the placebo group. However testosterone levels were significantly higher in the treatment group in the second (*t*(239) = −18.61, *p* < 0.001), third (*t*(239) = −24.70, *p* < 0.001) and fourth (*t*(239) = −25.80, *p* < 0.001) saliva sample, providing a robust and successful manipulation check to our pharmacological testosterone treatment. Mean (SD) non-logged testosterone levels 11,342.27 (15,270.73)pg/mL in treatment and 249.00 (274.20)pg/mL in placebo at the second saliva sample, 20,609.34 (20027.17)pg/mL in treatment and 353.36 (570.76)pg/mL in placebo at the third saliva sample, and 9.16 (1.40)pg/mL in treatment and 5.19 (0.92)pg/mL in placebo at the fourth saliva sample. These changes in salivary testosterone levels are in line with other studies which also used topical testosterone and progesterone administration^60,61^. There were no treatment effects on either mood, treatment expectancy, or levels of all other measured hormones, ruling out these potential indirect treatment influences on the task (see Supplementary Materials Table S4 for further details).

### The Influence of Testosterone Administration on Deception

We use three non-parametric measures to compare the two treatment groups: the distribution of rolls via a *χ*^2^-test against equal distributions, the mean reported roll via a Mann-Whitney U-test of differences, and the reported proportion of the highest possible roll via a Fisher’s exact test.

**Figure 1:**
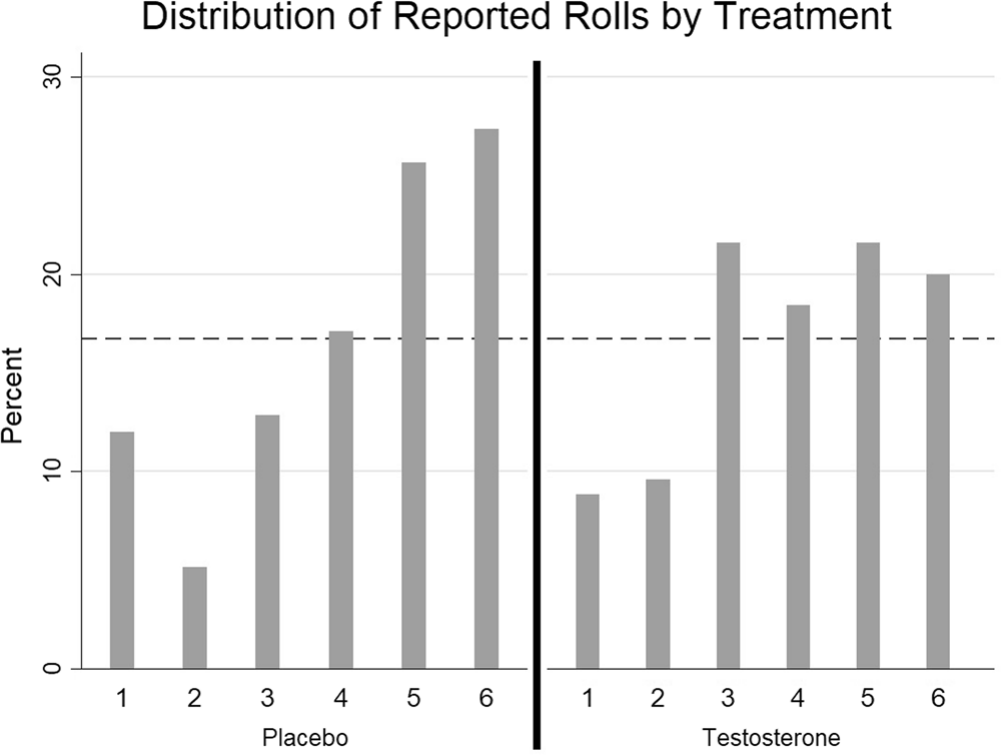
Distribution of reported rolls by treatment. Reference line is the expected frequency of each outcome with fully honest participants.

A *χ*^2^-test confirms that both treatment groups exhibited evidence of self-serving lying, as indicated by a right-skewed distribution (*χ*^2^-test of even distribution, Testosterone *χ*^2^(5) = 26.71, *p* = 0.02, Placebo *χ*^2^(5) = 13.10, *p* < 0.001, see Fig. 1). This common use of lying is in line with other research using the die-roll task^4,10,55,62^ and demonstrates that participants grasp that they are able to misreport their rolls presumably in order to increase their earnings and do so. A Mann-Whitney U-test of differences in the distributions of reported roll yielded could not reject the null that the distributions are the same (*z*(240) = 1.57, *p* = 0.12). The Fisher’s exact test could not reject the null that the proportion of 6’s reported in each treatment group are the same (Fisher’s exact, *p* = 0.17).

**Figure 2:**
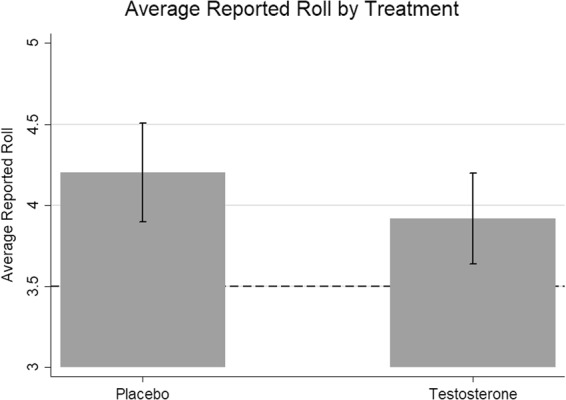
Average reported roll by treatment. Reference line is the expected average roll with fully honest participants. Bars represent 95% CI.

We also report the result of a parametric t-test, and use this summary statistic in order to perform a joint analysis of our study together with the results of Wibral *et al*. Overall, our findings are similar, regardless of whether we use a parametric or non-parametric approach. The average reported die roll in our sample of the placebo group was 4.21 (95% CI[3.91, 4.52]) and treatment group was 3.94 (95% CI[3.67, 4.22]), which by a 2-sided t-test did not significantly differ (*t*(240) = −1.31, *p* = 0.19, Cohen’s *d* = −0.17, 95% CI[−0.42, 0.08], see Fig. 2).

### Comparison to Wibral *et al*

In Table 2 we juxtapose the major statistical results from our study and those from Wibral *et al*.^45^. Overall, our results are directionally the same in that testosterone is associated with a decrease in reported rolls, but we do not find statistical significance by any measure at the 10% level. The key reported measures of Wibral *et al*. were the Mann-Whitney U-test of different distributions between treatment groups (*z*(89) = 2.78, *p* = 0.01) and the Fisher’s exact test of different frequencies of reporting the number with the highest material incentive (*p* = 0.01), which we contrast with our Mann-Whitney U-test result (*z*(240) = 1.57, *p* = 0.12), and Fisher’s exact test result (*p* = 0.18).

**Table 2:** Comparisons of major statistical findings with Wibral *et al*.

Statistical Test	Description	Wibral *et al*.	Current Study
Mann-Whitney U-test	Comparison of mean post-treatment testosterone levels between groups	*N* = 91, *p* = 0.03	*N* = 241, *z*(239) = −12.79, *p* < 0.001
*χ*^2^-test against uniform distribution	Test for evidence of self serving lying, i.e. for a right-skewed distribution in the reported die rolls	Treatment *N* = 46, *χ*^2^(5) = 13.22, *p* = 0.02 Placebo *N* = 45, *χ*^2^(5) = 63.47, *p* < 0.001	Treatment *N* = 125, *χ*^2^(5) = 13.10, *p* = 0.02 Placebo *N* = 117, *χ*^2^(5) = 26.71, *p* < 0.001
Mann-Whitney U-test	Test for differences in the distributions of reported die rolls between testosterone and placebo	*N* = 91, *z*(89) = 2.78, *p* = 0.01	*N* = 242, *z*(240) = 1.57, *p* = 0.12
Fisher’s exact test	Test whether the number with the highest payoff was reported more frequently in treatment as compared to control (in Wibral *et al*. it was the number 5, in our study it is the number 6)	Proportion of 5’s in treatment 16/46 = 35% Proportion of 5’s in placebo 28/45 = 62% *p* = 0.01, *n* = 91	Proportion of 6’s in treatment 25/125 = 20% Proportion of 6’s in placebo 32/117 = 27% *p* = 0.17, *n* = 242
2 sided T-test	Comparison of the mean reported roll between testosterone and placebo groups	Mean (SD) treatment = 3.33 (1.67) Mean (SD) placebo = 4.18 (1.37) *t*(89) = 2.65, *p* < 0.001	Mean treatment (SD) = 3.94 (1.39) Mean placebo (SD) = 4.21 (1.66) *t*(240) = 1.31, *p* = 0.19

In terms of effect size, Wibral *et al*. found a medium effect size of Cohen’s *d* = −0.56 (95% CI[−0.97, −0.14]) of the impact of testosterone on average reported die-roll. Based on this effect size, a sample size of 81 would be sufficiently powered at *β* = 0.80 at the 5% level. A typical finding in the replication literature is that the replicated effect size is smaller than the original by about a half in psychological experiments^47^ and a third in experimental economics^48^. With our sample size of N = 242, we achieved *β* = 0.88 at the 5% level for detecting the 2/3 of the original effect size, or *β* = 0.68 at the 5% level for detecting one half of the original effect size. Our small effect size of Cohen’s *d* = −0.17 (95% CI[−0.42, 0.08]) suggests that we would have needed a sample size of *N* = 878 to detect a significant difference in means for the point estimate, and *N* = 142 to detect the upper bound of our confidence interval at the 5% level with *β* = 0.80.

### Joint Analysis of Studies

**Figure 3:**
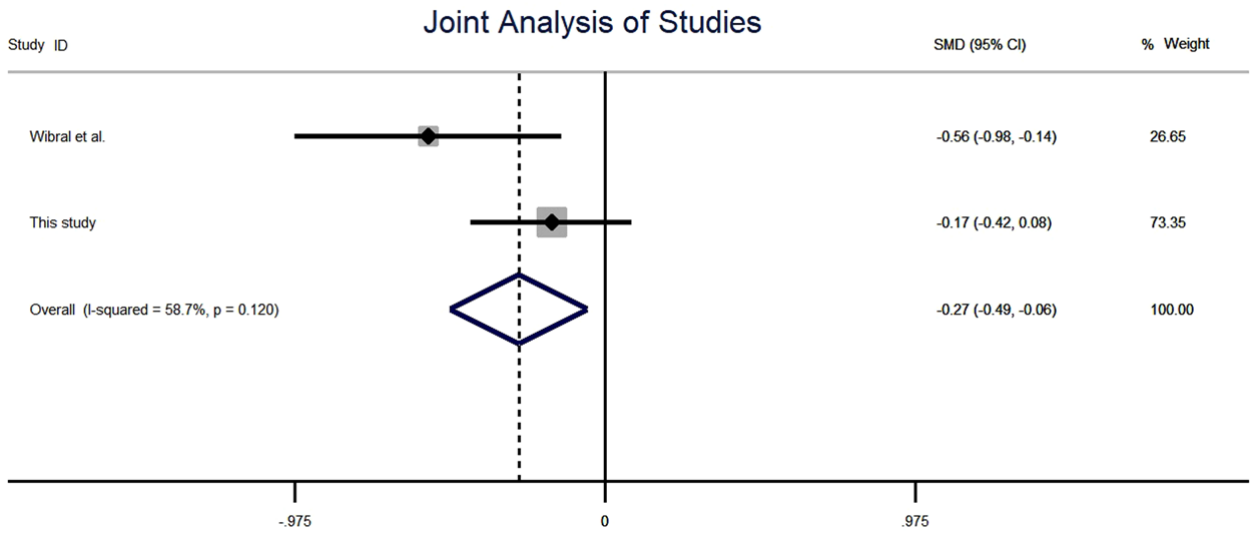
Meta-analysis of effect size using fixed effects model. Bars represent 95% CI.

To perform the joint analysis we use a fixed effects model using a weighted average of both studies, in line with previous work on replications^47,48^. Because the system of payoffs in the Wibral *et al*. study was such that reporting a 6 earned nothing, we transformed their data to match our own based on the payoffs associated with each report, such that a report of 5 was coded as 6, 6 was coded as 1, 1 was coded as 2, *et cetera*. Using the fixed effects model, Cohen’s *d* is equal to −0.27 (95% CI[−0.49, −0.06]) and a test of *d* = 0 is rejected at the 0.05 level (*z*(1) = 2.46, *p* = 0.01, see Fig. 3). The achieved power is >0.999, calculated using G-Power.

Further details, including robustness to a random effects specification and a search for comparable studies are in the Supplementary Materials.

### Effect of Winning or Losing in Risk Task

As discussed previously in Differences From Wibral *et al*., this task was part of an experimental battery, and the preceding task was a risk task in which participants were divided into winners and losers based on their performance. In order to test an association between the competition outcomes and a potential interaction between competition outcomes and treatment, we ran a two-way ANOVA with competition outcomes (winning/losing) and treatment (testosterone/placebo) as between-subject factors, as well as an interaction term. We found no significant effects of competition outcome on reported die roll (*F*(1, 234) = 0.71, *p* = 0.401), treatment condition (*F*(1, 234) = 3.21, *p* = 0.074]), or interaction (*F*(1, 234) = 1.41, *p* = 0.236).

### Facial Masculinity and Digit Ratio

To test for the impact of digit ratio and facial masculinity on behavior, we performed a number of ordinary least squares regressions. Regressing die roll on treatment, digit ratio, and the interaction of digit ratio and treatment did not yield any coefficients significantly different from 0 (treatment *β* = −5.159(5.835), 95% CI[−16.654, 6.335], *t*(235) = −0.88, *p* = 0.377, digit ratio *β* = −4.278(4.352), 95% CI[−12.851, 4.294], *t*(235) = −0.98, *p* = 0.327, interaction *β* = 5.147(6.149), 95% CI[−6.966, 17.260], *t*(235) = 0.84, *p* = 0.403). Similarly, regressing die roll on treatment, facial masculinity, and the interaction of treatment and facial masculinity did not yield any coefficients significantly different from 0 (treatment *β* = −0.018(0.644), 95% CI[−1.287, 1.251], *t*(227) = −0.03, *p* = 0.978, facial masculinity *β* = 0.160(0.282), 95% CI[−0.396, 0.715], *t*(227) = 0.57, *p* = 0.572, interaction *β* = −0.126(0.390), 95% CI[−0.895, 0.643], *t*(227) = −0.32, *p* = 0.746). The results remained insignificant when including measurements of other hormonal levels and demographic characteristics, as reported in the Supplementary Materials.

## Discussion

The present study found modest evidence for testosterone reducing self-serving dishonesty. Although statistically insignificant, the direction was in same direction of the original study, with our joint analysis indicating a significant effect (*p* = 0.01) of testosterone administration on the mean die roll report. It should be taken as suggestive, but not conclusive, evidence of a relationship between testosterone and reduced lying, that should encourage further exploration. In this section we discuss the limitations of our study, and then suggest avenues for future research elaborating on the association between testosterone and deception.

Despite the advantages of strict experimental control, there are several limitations inherent in the methodology of the current study.

First, a general limitation of laboratory studies is that at the participants are aware that they are taking part in an experiment. One cannot entirely rule out the possibility that such knowledge might bias behavior (e.g., via experimenter demand effect), in a way that could interact with the treatment.

A second limitation of the particular task at hand (where we do not directly observe the behavior of the participants), is the incapacity to measure whether or by how much each individual participant lied. This limits, to some degree, the capacity to explore which factors might moderate of the behavioral effect.

Third, while the use of college students in scientific experiments, particularly the behavioral sciences, is a widely accepted practice, it comes with specific considerations to be made when generalizing findings to other populations. The possibility of an interaction between our subject population characteristics (young males) and our experimental design is particularly relevant, as the levels of testosterone decrease with age after 20^63^ and vary significantly between sexes^64^. Thus, our sample is not representative of the baseline physiology of the general population. Furthermore, the proposed psychological mechanism through which testosterone impacts lying is through social-status concerns, and it may be that different demographic groups would not pursue social status goals through honesty on this task. Therefore, we advise that any generalized interpretation of our findings to other populations should be made with caution.

Going forward, elucidating the relationship between testosterone and deception requires clear hypotheses of connecting mechanisms and methodologies that directly test them. The relatively complex chain of reasoning connecting testosterone and deception in the die-roll task proposed by Wibral *et al*. is that testosterone increases status seeking, and thus elevates the decision maker’s need for pride, which in turn promotes honest behavior. However, as deception is typically associated with material benefits that are also important for one’s social status, it is not *a priori* clear whether testosterone-induced status seeking should decrease, rather than increase honesty in this task. Using deception tasks with more obvious social status interpretations would provide a stronger test of this potential connection.

Another potential mechanism by which testosterone impacts die roll reports may be through its influence on impulsivity^31^. Greater impulsivity may reduce the propensity of an individual to engage in processes which either increase or decrease their ultimate willingness to lie. For example, reflection could either increase lying by justifying it as harming no one^10^, or decrease it by reflecting upon moral considerations. Further experimental work that aspires to explore this issue should have clear predictions about whether lying in the specific behavioral task used is more associated with either impulsive or deliberative decision-making.

A final possibility is that testosterone may increase the feelings of distrust in participants. Several studies have found a negative relationship between testosterone administration and trust, as measured by reduced offers in the trust game^36^ and facial trustworthiness evaluations by women^65^. Moreover, studies of anabolic steroid users found that they are more likely to report paranoia, even after short-term use^66–68^. Even though both in our study and in Wibral *et al*. the researchers made efforts to provide privacy for the participants and ensure them of this fact, recent research suggests that lying in the die-roll task is partly driven by fears of detection^5^. Thus, increased feelings of distrust might lead participants to doubt that the researchers were truly unable to observe their actions, or to be concerned of a hidden or unstated punishment for being observed deceiving. Further research could attempt to address this issue by including survey measures to assess whether or not participants felt as if their actions were truly performed in privacy if applicable, or using methodologies where a lie is completely undetectable. An example of such a methodology is a “mind” game in which participants think of a number and then roll a die in private, and report whether the rolled number matched the number they thought of, which was used in Kajackaite and Gneezy^5^.

In summation, we find a statistically insignificant negative effect of testosterone administration on mean reported die roll. When jointly considered along with results from a previous and similar study by Wibral *et al*., there is overall evidence of a negative association between testosterone and lying. There are a number of plausible mechanisms which might explain this association, but currently with data only from the die-roll task it is not possible to determine which mechanism(s) play a central role. In addition to designing future studies around straightforward tests of these mechanisms, researchers should use large sample sizes and facilitate the replications of their findings. Evidence is growing that testosterone impacts behavior in diverse ways, and practices which help build a robust knowledge base on these impacts is paramount for progress.

## Electronic supplementary material

Supplementary Materials

## References

[1] Gneezy U (2005). Deception: The role of consequences. Am. Econ. Rev..

[2] Gächter S, Schulz JF (2016). Intrinsic honesty and the prevalence of rule violations across societies. Nature.

[3] Mann H, Garcia-Rada X, Hornuf L, Tafurt J, Ariely D (2016). Cut from the same cloth: Similarly dishonest individuals across cultures. J. Cross. Cult. Psychol..

[4] Fischbacher U, Franziska F (2013). Lies in disguise—an experimental study on cheating. Journal of the European Economic Association.

[5] Kajackaite A, Gneezy U (2017). Incentives and cheating. Games. Econ. Behav..

[6] Charness G, Masclet D, Villeval M (2014). The dark side of competition for status. Manage. Sci..

[7] Becker, G. S. Crime and punishment: An economic approach. *The Economic Dimensions of Crime*. 13–68 (1968).

[8] Schurr A, Ritov I (2016). Winning a competition predicts dishonest behavior. Proceedings of the National Academy of Sciences.

[9] Shalvi S, Dana J, Handgraaf MJJ, De Dreu CKW (2011). Justified ethicality: Observing desired counterfactuals modifies ethical perceptions and behavior. Organ. Behav. Hum. Decis. Process..

[10] Shalvi S, Eldar O, Bereby-Meyer Y (2012). Honesty requires time (and lack of justifications). Psychol. Sci..

[11] Mazar N, Amir O, Ariely D (2008). The dishonesty of honest people: A theory of self-concept maintenance. J. Mark. Res..

[12] Dreber A, Johannesson M (2008). Gender differences in deception. Econ. Lett..

[13] Muehlheusser G, Roider A, Wallmeier N (2015). Gender differences in honesty: Groups versus individuals. Econ. Lett..

[14] Trivers R (2000). The elements of a scientific theory of self-deception. Ann. N. Y. Acad. Sci..

[15] Bugnyar T, Kotrschal K (2002). Observational learning and the raiding of food caches in ravens, corvus corax: is it ‘tactical’ deception?. Anim. Behav..

[16] Whiten A, Byrne RW (1988). Tactical deception in primates. Behavioral & Brain Sciences.

[17] Volz KG, Vogeley K, Tittgemeyer M, von Cramon DY, Sutter M (2015). The neural basis of deception in strategic interactions. Front. Behav. Neurosci..

[18] Lisofsky N, Kazzer P, Heekeren HR, Prehn K (2014). Investigating socio-cognitive processes in deception: a quantitative meta-analysis of neuroimaging studies. Neuropsychologia.

[19] Spence S (2004). A cognitive neurobiological account of deception: evidence from functional neuroimaging. Philos. Trans. R. Soc. Lond. B Biol. Sci..

[20] Garrett N, Lazzaro SC, Ariely D, Sharot T (2016). The brain adapts to dishonesty. Nat. Neurosci..

[21] Shalvi S, De Dreu CKW (2014). Oxytocin promotes group-serving dishonesty. Proceedings of the National Academy of Sciences.

[22] Pfundmair M, Erk W, Reinelt A (2017). Lie to me—oxytocin impairs lie detection between sexes. Psychoneuroendocrinology..

[23] Lane, A., Luminet, O., Nave, G. & Mikolajczak, M. Is there a publication bias in behavioural intranasal oxytocin research on humans? Opening the file drawer of one laboratory. *Journal of Neuroendocrinology* **28** (2016). 10.1111/jne.12384 26991328

[24] Walum H, Waldman I, Young LJ (2016). Statistical and methodological considerations for the interpretation of intranasal oxytocin studies. Biol. Psychiatry.

[25] Nave G, Camerer CF, McCullough M (2015). Does oxytocin increase trust in humans? A critical review of research. Perspectives on Psychological Science.

[26] Pope HG, Kouri EM, Hudson JI (2000). Effects of supraphysiologic doses of testosterone on mood and aggression in normal men: a randomized controlled trial. Arch. Gen. Psychiatry.

[27] Wang CEA (2004). Long-term testosterone gel (androgel) treatment maintains beneficial effects on sexual function and mood, lean and fat mass, and bone mineral density in hypogonadal men. The Journal of Clinical Endocrinology & Metabolism.

[28] Gray PEA (2005). Dose-dependent effects of testosterone on sexual function, mood, and visuospatial cognition in older men. The Journal of Clinical Endocrinology & Metabolism.

[29] Newman ML, Sellers JG, Josephs RA (2005). Testosterone, cognition, and social status. Horm. Behav..

[30] Janowsky JS, Oviatt SK, Orwoll ES (1994). Testosterone influences spatial cognition in older men. Behav. Neurosci..

[31] Nave G, Nadler A, Zava D, Camerer C (2017). Single-dose testosterone administration impairs cognitive reflection in men. Psychol. Sci..

[32] Apicella CL, Carré JM, Dreber A (2015). Testosterone and economic risk taking: A review. Adaptive Human Behavior and Physiology.

[33] Nadler, A., Jiao, P., Alexander, V., Johnson, C. J. & Zak, P. J. The bull of wall street: Experimental analysis of testosterone and asset trading. *Management Science* (Forthcoming).

[34] Coates JM, Herbert J (2008). Endogenous steroids and financial risk taking on a london trading floor. Proceedings of the National Academy of Sciences.

[35] Cueva C (2015). Cortisol and testosterone increase financial risk taking and may destabilize markets. Sci. Rep..

[36] Boksem MA (2013). Testosterone inhibits trust but promotes reciprocity. Psychol. Sci..

[37] Van Honk, J., Montoya, E. R., Bos, P. A., van Vugt, M. & Terburg, D. New evidence on testosterone and cooperation. *Nature* **485**, 7399 (2012). 10.1038/nature11136 22622587

[38] Eisenegger C, Naef M, Snozzi R, Heinrichs M, Fehr E (2010). Prejudice and truth about the effect of testosterone on human bargaining behaviour. Nature.

[39] Hermans EJ, Ramsey NF, van Honk J (2008). Exogenous testosterone enhances responsiveness to social threat in the neural circuitry of social aggression in humans. Biol. Psychiatry.

[40] Carré, J. M., Ruddick, E. L., Moreau, B. J. & Bird, B. M. *Testosterone and Human Aggression*. (2017).

[41] Eisenegger C, Haushofer J, Fehr E (2011). The role of testosterone in social interaction. Trends. Cogn. Sci..

[42] Dreher J (2016). Testosterone causes both prosocial and antisocial status-enhancing behaviors in human males. Proceedings of the National Academy of Sciences.

[43] Nave, G. *et al*. Single-dose testosterone administration increases men’s preference for status goods. *Nature Communications* (Forthcoming). 10.1038/s41467-018-04923-0 PMC6030157 29970895

[44] Van Honk J (2016). Effects of testosterone administration on strategic gambling in poker play. Sci. Rep..

[45] Wibral M (2012). Testosterone administration reduces lying in men. PLoS. ONE..

[46] Dai, Z., Galeotti, F. & Villeval, M. C. Cheating in the lab predicts fraud in the field: An experiment in public transportation. *Management Science* (2017).

[47] Open Science Collaboration. Estimating the reproducibility of psychological science. *Science* **349**, 6251 (2015). 10.1126/science.aac4716 26315443

[48] Camerer CF (2016). Evaluating replicability of laboratory experiments in economics. Science.

[49] Zethraeus N (2009). A randomized trial of the effect of estrogen and testosterone on economic behavior. Proceedings of the National Academy of Sciences.

[50] Simonsohn U (2015). Small telescopes: Detectability and the evaluation of replication results. Psychol. Sci..

[51] Lutchmaya S, Baron-Cohen S, Raggatt P, Knickmeyer R, Manning JT (2004). 2nd to 4th digit ratios, fetal testosterone and estradiol. Early Hum. Dev..

[52] Penton-Voak IS, Chen JY (2004). High salivary testosterone is linked to masculine male facial appearance in humans. Evol. Hum. Behav..

[53] Eisenegger C, von Eckardstein A, Fehr E, von Eckardstein S (2013). Pharmacokinetics of testosterone and estradiol gel preparations in healthy young men. Psychoneuroendocrinology..

[54] Chik Z (2006). Pharmacokinetics of a new testosterone transdermal delivery system, tds®-testosterone in healthy males. Br. J. Clin. Pharmacol..

[55] Abeler, J., Nosenzo, D. & Raymond, C. Preferences for truth-telling. *IZA Discussion**Paper 10188* (2016).

[56] Slovic P (1966). Risk-taking in children: Age and sex differences. Child Dev..

[57] Montoya E, Terburg D, Bos PA, van Honk J (2012). Testosterone, cortisol, and serotonin as key regulators of social aggression: A review and theoretical perspective. Motiv. Emot..

[58] DiMaggio P (1982). Cultural capital and school success: The impact of status culture participation on the grades of us high school students. Am. Sociol. Rev..

[59] Hugh-Jones, D. Honesty and beliefs about honesty in 15 countries. *University of East Anglia Discussion Paper* (215).

[60] Mayo A, Macintyre H, Wallace A, Ahmed S (2004). Transdermal testosterone application: pharmacokinetics and effects on pubertal status, short-term growth, and bone turnover. The Journal of Clinical Endocrinology & Metabolism.

[61] Du JY (2013). Percutaneous progesterone delivery via cream or gel application in postmenopausal women: a randomized cross-over study of progesterone levels in serum, whole blood, saliva, and capillary blood. Menopause.

[62] Erat S, Gneezy U (2012). White lies. Manage. Sci..

[63] Harman SMEA (2001). Longitudinal effects of aging on serum total and free testosterone levels in healthy men. The Journal of Clinical Endocrinology & Metabolism.

[64] Torjesen PA, Sandnes L (2004). Serum testosterone in women as measured by an automated immunoassay and a ria. Clin. Chem..

[65] Bos PA, Terburg D, van Honk J (2010). Testosterone decreases trust in socially naive humans. Proceedings of the National Academy of Sciences.

[66] Perry PJ, Anderson KH, Yates WR (1990). Illicit anabolic steroid use in athletes. a case series analysis. Am. J. Sports Med..

[67] Pope HG, Katz DL (1988). Affective and psychotic symptoms associated with anabolic steroid use. Am. J. Psychiatry.

[68] Wilson I, Prange A, Lara P (1985). Methyltestosterone and imipramine in men: conversion of depression to a paranoid reaction. Am. J. Psychiatry.

