# Supplementary material for: Testing the influence of testosterone administration on men’s honesty in a large laboratory experiment
Source: Sci Rep. 2018 Aug 1;8:11556. doi: 10.1038/s41598-018-29928-z (PMC6070559; doi:10.1038/s41598-018-29928-z)
Supplement: Supplementary file 1 — Supplementary Materials [file 41598_2018_29928_MOESM1_ESM.pdf]

Supplementary Materials for

Testing the influence of testosterone administration on men's honesty in a large laboratory experiment

Austin Henderson, Garrett Thoelen, Amos Nadler, Jorge A. Barraza, Gideon Nave

Table of Contents

1. Participants 2

2. Methods 4

3. Hormonal assay procedure 5

4. Hormonal changes following treatment and manipulation check 7

5. Measuring Digit Ratio and Facial Masculinity 10

6. Behavioral task batteries 13

7. Regression analyses 14

8. Joint analysis discussion 19

9. References 20

1. Participants

There were N = 242 male participants. Most (217, 90%) were students from a southern Californian college. Non-student participants were community members from surrounding cities. N = 125 of participants were randomly assigned to receive a standard dose of transdermal testosterone (abbreviated "T") gel, and N = 117 received placebo gels of matched viscosity in a double blind exogenous administration paradigm.

Pre-screening criteria excluded everyone with relevant medical and psychological conditions (5α-reductase deficiency, Klinefelter's syndrome, brain tumor, cancer, psychiatric diagnosis/diagnoses, high blood pressure, liver disease, kidney disease, angina, cancer, hepatitis, renal/kidney impairment, history of epileptic seizures and hypersensitivity to soy/ alcohol), participants using prescription drugs that may interfere with the study (oxyphenbutazone, insulin, corticosteroids, opioids), participants who self-reported consuming illegal drugs or excessive alcohol in the last 24 hours and non-native English speakers.

Personal, demographic, and treatment expectancy characteristics of the two treatment groups are summarized in Table S1 (note that 5 participants did not report their age and were therefore excluded from all analyses in which age is used as a control variable). The right column of Table S1 also reports the *p*-value of two-sample t-tests for differences between T and placebo group characteristics (a check on whether random assignment resulted in balance on all such variables). Two participants (one from each treatment group) self-reported taking T treatment on a regular basis; all analyses include these participants and are robust to excluding them.

Table S1: Self-reported demographic data summary (standard errors in parentheses)

	All	T	Placebo	*P*-values for test of difference
**N**	242	125	117	
**Age**	23.65<br>(0.47)	24.42<br>(0.77)	22.81<br>(0.50)	0.09
**Left-handed (proportion)**	0.078<br>(0.02)	0.072<br>(0.02)	0.085<br>(0.03)	0.70
**Heterosexual (proportion)**	0.06<br>(0.02)	0.07<br>(0.02)	0.05<br>(0.02)	0.51
**Treatment expectancy**[1]	2.78<br>(0.06	2.68<br>(0.08)	2.88<br>(0.09)	0.11
**Married (proportion)**	0.08<br>(0.02)	0.09<br>(0.03)	0.08<br>(0.02)	0.76
**In a relationship (proportion)**	0.41<br>(0.03)	0.38<br>(0.05)	0.45<br>(0.05)	0.25
**Has children**	0.06<br>(0.02)	0.08<br>(0.02)	0.03<br>(0.02)	0.12
**Personal monthly income**[2]	2.05<br>(0.11)	2.02<br>(0.14)	2.08<br>(0.16	0.80

1 5 point scale, 1 = definitely did not get T, 2 = probably not, 3 = unsure, 4 = probably got T, 5 = definitely got T
2 5 point scale, 1 = under $500/month, 2 = $501-$1,000, 3 = $1,001-$1,500/month, 4 = $1,501-$2,000/month 5 = over $2001/month

2. Methods

Instructions were given using Qualtrics (Figure S1), an online survey platform commonly used for academic research, which was also used in other parts of the experiment.

Figure S1. Instructions for the die-roll task.

You now have the chance to receive an additional payoff by rolling a die and reporting the outcome.
The amount of money you earn will be determined by a single roll of the die located on your desk.
The experimenter does not control the number you disclose.

Instructions:
1. Inspect the die and verify it is six-sided and contains all numbers 1 through 6.
2. Roll the die once on the table in front of you.
3. Type the number of dots facing upwards on the die in the screen below. This is the amount of money you'll receive for this portion.

Example: You roll the die and the result was 3. Type the number 3 in the box; you will earn an additional $3 in your final payout.

Please check your die to ensure that it is 6-sided now. If you would like, you can throw the dice more than once, however, only the first throw counts.

If you have questions, please raise your hand.

Otherwise, roll the die and state the outcome below **and in your payment form (the one you have just received), in the box below "Dice Roll"**.

>>

Participants in the die roll task were asked to record and submit the number that they rolled through 2 forms: by entering the number into the appropriate field on Qualtrics (the method used for primary data analysis), and by writing on a slip of paper which was then submitted to a research assistant. The reason for using two methods was that the hand-written slips facilitated quickly calculating the payoffs for participants. A later review to check for discrepancies between the two data sources revealed that some receipts (seven) had been lost and (nine) were damaged while submitted along with other financial documents for reimbursement. Of the overlapping 226 data points between those entered on Qualtrics and those written by hand, only four participants submitted different die rolls. Of those, two reported a higher roll on Qualtrics relative to hand-written and 2 conversely reported a lower roll. Running the exact same analysis on the data set of hand-written responses did not yield any differences from Qualtrics data; all of the conclusions of the paper are robust to either data set.

3. Hormonal assay procedure

Salivary steroids (estrone, estradiol, estriol, T, androstenedione, DHEA, 5- alpha DHT, progesterone, 17OH-progesterone, 11-deoxycortisol, cortisol, cortisone, and corticosterone) were measured by liquid chromatography tandem mass spectrometry (LC-MS/MS) using an AB Sciex Triple Quad 5500. Internal standards were added to 1 ml of saliva and the steroids then extracted by C18 column chromatography with 0.1 M NH4OH wash followed by 10% acetone. Steroids were eluted from the SPE with 10% methanol in acetone and dried under nitrogen. The dried samples were subjected to derivatization—the process of transforming a compound into a derivative product of similar chemical structure—with pyridine-3-sulfonyl chloride for the estrogens (estrone (E1), estradiol (E2), and estriol (E3)) as outlined by Xi and Spink (2008). 40 µL sodium bicarbonate (50mM, pH 10) and 40 µL pyridine-3-sulfonyl chloride (3 mg/mL in acetonitrile) were added to the dried samples, and incubated at 60°C for 10 minutes. After derivatization, the samples were diluted with 80 µL of water and injected for LC-MS/MS analysis with analytical separation performed on an Agilent Poroshell 120 EC-C8 column and ionization by atmospheric pressure chemical ionization (APCI) in the positive ionization mode. Table S2 lists each analyte along with its validation results for the Lower Limit Of Quantification (LLOQ) for the lowest level of detection with coefficients of variation (CVs) < 20% over the linear range, linear range, and the inter-assay precision from the highest concentration to the LLOQ within the linear range. When salivary hormone levels of participants were below their LLOQ, we assigned values halfway between zero and their respective LLOQ (note that the true quantities of the hormone in the sample are never zero, even when they do not reach the detection threshold).

Table S2: Detection levels, precision and normality tests of hormonal assay

Analyte	LLOQ	Range	Precision	Proportion undetected, pre-treatment sample A	Proportion undetected, first post-treatment sample B	K-S test *p*-value	K-S test (log) *p*-value
**Estrone** pg/mL	0.5	0.5 - 510	8.7 - 13.7%	0.132	0.257	<0.01	0.56
**Estradiol** pg/mL	0.3	0.3 - 510	4.3 - 18.7%	0.128	0.329	0.06	0.88
**Testosterone** pg/mL	3.0	3.0 - 5100	3.0 - 18.1%	0	0.008	$<10^{-20}$	<0.01
**Androstenedione** pg/mL	5.0	5.0 - 2300	5.2 - 6.6%	0	0.008	$<10^{-20}$	0.008
**DHEA** pg/mL	20.0	20.0 - 1800	4.1 - 15.2%	0.004	0.012	0.002	0.98
**DHT** pg/mL	10.0	10.0 - 920	3.6 - 17.7%	0.786	0.473	$<10^{-11}$	0.02
**Progesterone** pg/mL	10.0	10.0 - 10000	4.8 - 10.8%	0.794	0.753	<0.01	0.03
**17OH-Progesterone** pg/mL	5.0	5.0 - 630	3.9 - 13.8%	0.004	0.061	0.003	0.98
**11-Deoxycortisol** pg/mL	5.0	5.0 - 410	6.8 - 16.6%	0.132	0.473	<0.01	0.04
**Cortisol** ng/mL	0.1	0.1 - 52	5.1 - 17.9%	0	0.008	<0.01	0.92
**Cortisone** ng/mL	0.1	0.1 - 81	4.1 - 14.9%	0	0.008	0.07	0.59
**Corticosterone** pg/mL	5.0	5.0 - 1500	4.6 - 17.5%	0.313	0.312	<0.01	0.08
**Aldosterone** pg/mL	10.0	10.0 - 650	8.9 - 18.8%	0.272	0.272	<0.06	0.39
**Melatonin** pg/mL	2.5	2.5 - 10000	5.2 - 15.9%	0.502	0.500	0.07	0.14

Note: *p*-values are calculated using a Kolmogorov-Smirnov test for the distributions of the second saliva sample compared to Gaussian, and for the log-transform (the null hypothesis is normal Gaussian distribution).

4. Hormonal changes following treatment and manipulation check

As expected, there were significant post-treatment differences between treatment groups with respect to all hormones influenced by T treatment, either as an upstream (androstenedione) or downstream (5-alpha DHT) metabolite of T (see Table S3). There was also a decrease in progesterone 170H resulting from an increase in T. The changes in saliva T measures were similar in magnitude to those reported in previous studies following topical gel administration of T and progesterone, e.g. (S2, S3).

We observed no significant differences between treatment groups in hormones that were not expected to change following short-term T treatment (e.g., aldosterone, cortisol, cortisone, melatonin) in all four saliva measurements throughout the experiment (i.e., the pre-treatment and the three post-treatment measurements, with two-tailed T-tests of equality showing all *p*-values > 0.20). The pre-treatment and first post-treatment mean hormonal saliva levels are summarized in table S3; note that differences between morning and afternoon hormonal levels were affected by diurnal cycles in both treatment groups.

From assays conducted during the first 13 (out of 17) sessions of the study, we noted that 72 out of 184 pre-treatment baseline saliva samples (in both treatment groups) presented measurements with higher T level that are expected in normal young men (greater than 400 pg/mL). All other measurements (including T metabolites) were hormonally typical. The effects of T on die roll were robust to excluding the participants with abnormal measurements (see section 6 below).

We traced the cause of these abnormal measurements to T gel transfer to common surfaces (e.g., door knobs, mouse pads). Crucially, the high measurements were caused by local spread of T into saliva tubes, but physiological levels were unaffected by superficial contact with the dry nuisance T gel, as (a) we observed normal pre-treatment levels of T metabolites, namely DHT and androstenedione in all participants; (b) none of the placebo group participants showed abnormally high values of T metabolites in any of the post-treatment measurements; (c) Only five out of 118 participants from the placebo group showed consistently elevated T measurements in all of the 3 post-treatment saliva samples; (d) previous investigations found that interpersonal T transfer is highly unlikely even with skin-to-skin contact (S4). Thus, we found convergent evidence that biofluid levels were unaffected by superficial contact. This conclusion was supported by ZRT Laboratories chief scientist Dr. David Zava.

In response to this finding during the course of the experimental period, we identified all surfaces and objects through which T could spread in the facility and improved sterile isolation protocol to eliminate the spread of the dried T gel. This protocol included thorough cleaning of keyboards, computer mice, chair backs, displays, and all door knobs with a bleach-alcohol solution after each session as well as asking participants to carefully wipe hands with a wet tissue before collecting each saliva sample. New pens were used for each session while all previously used pens were removed from testing area. Clipboards and other miscellaneous objects that participants did or could interact with were cleaned, and an aerosol "air sanitizer" that bonds to volatile organic compounds was sprayed into the air. Following the adoption of this strict

sterilization protocol, we found a drastic reduction in incidence of high T samples in the pre-treatment measurements, to a total of 5 participants out of 58 in the following four sessions (sessions 14-17).

Table S3: hormone panel data measurements log(pg/mL) summary statistics (standard errors in parentheses)

	**Placebo**		**Testosterone**		**Two-tailed *p*-value from t-test of T-Placebo equality**	
**Sampling time**[1]	**9am**	**2pm**	**9am**	**2pm**	**9am**	**2pm**
**Testosterone**	**5.743 (0.094)**	**5.111 (0.085)**	**5.580 (0.084)**	**8.373 (0.151)**	**0.267**	**$1.06*10^{-13}$**
**Androstenedione**	**4.510 (0.039)**	**4.205 (0.044)**	**4.525 (0.034)**	**5.462 (0.084)**	**0.634**	**$3.11*10^{-9}$**
**DHT**	**1.984 (0.069)**	**1.867 (0.051)**	**1.905 (0.060)**	**3.482 (0.114)**	**0.745**	**$2.38*10^{-6}$**
Progesterone	1.937 (0.058)	2.002 (0.064)	1.829 (0.052)	1.883 (0.055)	0.36	0.41
**17OH-Progesterone**	**3.245 (0.050)**	**2.675 (0.058)**	**3.217 (0.049)**	**2.463 (0.058)**	**0.792**	**0.008**
Estrone	-0.088 (0.063)	-0.557 (0.066)	-0.007 (0.064)	-0.389 (0.056)	0.29	0.42
Estradiol	-0.743 (0.052)	-1.158 (0.059)	-0.766 (0.054)	-1.066 (0.054)	0.86	0.44
DHEA	5.198 (0.053)	4.570 (0.058)	5.116 (0.051)	4.557 (0.054)	0.30	0.76
11-Deoxycortisol	2.579 (0.079)	1.650 (0.072)	2.568 (0.083)	1.584 (0.064)	0.66	0.35
Cortisol	1.047 (0.058)	0.062 (0.065)	1.045 (0.057)	0.077 (0.058)	0.68	0.81
Cortisone	2.539 (0.030)	1.952 (0.060)	2.539 (0.034)	2.003 (0.050)	0.70	0.76
Corticosterone	2.442 (0.126)	1.274 (0.065)	2.646 (0.123)	1.290 (0.060)	0.37	0.76
Aldosterone	2.640 (0.067)	2.516 (0.071)	2.634 (0.068)	2.395 (0.066)	0.82	0.14
Melatonin	1.045 (0.093)	0.276 (0.029)	1.221 (0.101)	0.353 (0.051)	0.27	0.23

1 Main effects of time (afternoon vs treatment) are due to diurnal cycles of the hormones.

5. Measuring Digit Ratio and Facial Masculinity

Digit Ratio

In each morning session, participants had their hands scanned. This allowed for a straightforward determination of the length ratio between their 2nd and 4th digits (2D:4D). Scanned pictures of hands were processed by research assistants (RAs), who measured the number of pixels between the start and end of the 2nd and 4th digits, with the 2D:4D ratio then calculated by dividing the length of the 2nd digit by the length of the 4th. The pixel measurement was done using a software tool which counted the number of pixels between two manually selected points. The start of the finger was considered the crease at where it met the palm, the end was at the tip.

Two RAs measured each hand, and any discrepancies over 5% (detected automatically when entered into a spreadsheet) were rechecked by a senior researcher in order to ensure accuracy and precision.

Facial Masculinity

Below is the protocol used by RAs in order to make facial measurements used in facial masculinity analysis. RAs were given a photograph of each participant's face, which were taken in a standardized manner with even lighting and no obstructions (e.g. glasses). Measurements were taken by measuring the number of pixels between points on the face as per the protocol. The pixel measurement was done using a software tool which counted the number of pixels between two manually selected points. Two RAs measured each face according to the protocol, and any discrepancies over 5% (detected automatically when entered into a spreadsheet) were rechecked by a senior researcher in order to ensure accuracy and precision.

For further information on symmetry measurements, which were not used in this analysis, see Little et al. (2008), which was used as a training reference for RAs. For further information on fWHR measurements, see Lefevre et al. (2013), which was also used as a training reference for RAs.

Protocol used by RAs for Facial Measurement

Image Normalization
Images were normalized by inter-pupillary distance (IPD), 600 pixels
Tiddeman, Burt, and Perrett (2001)
http://facelab.org/bcjones/Teaching/files/Tiddeman_2001.pdf

1) Load image into Paint
2) Measure IPD, from the center of one pupil to the center of the other
3) Calculate the % change needed to resize the image to standardized IPD, 600 pixels (600/IPD)
4) Select-all
5) Resize, put number calculated in (3) into the horizontal box (make sure "Maintain aspect ratio" is still checked)

Symmetry Measurements

1) Establish vertical line of symmetry placing line of symmetry perpendicular to the inter-pupillary line at the midpoint.
2) Measure the distance between the line of symmetry and the point of measurement, e.g. midline-D3L (Outside of the cheekbone on the left-hand side of the image and midline-D3R (Outside of the cheekbone on the right-hand side of the image), and record these measurements in their appropriate cells in the "Symmetry" sheet of the Facial Measurements Google Spreadsheet
3) Repeat this for all symmetry relevant measurements (D1L-D6R)
4) The spreadsheet will automatically calculate the absolute value of the difference between the distance between the line of symmetry and the left and right measurements

Sexual Dimorphism (masculinity) measurements

1) Take measurements for sexual dimorphism (D7, D8, D9) according to figure above, and record measurements in their appropriate cell of the “Facial Masculinity” sheet in the Facial Measurements Google Spreadsheet.
2) (Jia et al. addendum)

Measure the vertical distance between the very top of the upper lip and the bottom of the upper eyelid (column titled VD)
Horizontal distance has already been measured (D3), so the second part of fWHR is already done.

---

6. Behavioral task batteries

The die roll task in both this study and in Wibral et al. was part of a larger battery of behavioral tasks in order to maximize the amount of data generated per pharmacological manipulation. Here are the experimental components in order from each study.

<u>Wibral et al.</u>
1. Risk and loss aversion
2. Selection between a fixed wage and tournament compensation for a simple calculation task
3. Eliciting a productivity measure for the calculation task
4. Discounting: 30 decisions between a smaller immediate reward and a larger delayed reward
5. Ultimatum Game
6. Overconfidence
7. Two-stage elimination tournament
8. Rating the attractiveness of female faces
9. Devil‘s Task
10. **Die roll**
11. Feedback on payoffs in the different experiments
12. Mental Rotation Test
13. Post-experimental questionnaire

<u>This study</u>
1. Status brands survey
2. Reading the mind in the eyes task
3. Competition (math task)
4. Auction
5. CRT
6. Risk taking
7. **Die roll**

7. Regression analyses

In order to test for the role of other covariates, we conducted several ordinary least squares regressions of other hormones, age, affect, treatment expectancy, and the ratio of the length of the 2nd and 4th fingers on average reported roll. We estimate the following 3 models, the results of which are reported in table S4.

A1 tests the effect of T treatment.

A2 tests the effect of T treatment, affect, age, treatment expectancy, digit ratio on the right hand, and facial width-height ratio.

A3 tests the above along with log transformed hormone levels at the final sample, which was nearest chronologically to the task.

We find no significant relationships between of any of these variables and reported die roll.

Table S4. Linear regression, dependent variable: reported die roll. Hormonal measurements are log transformed (standard errors in parentheses)

	(A1)	(A2)	(A3)
**Treatment**	-0.270<br>(0.206)	-0.331<br>(0.225)	-0.315<br>(0.237)
**Negative Affect**		-0.126<br>(0.213)	-0.119<br>(0.225)
**Positive Affect**		0.051<br>(0.120)	0.045<br>(0.123)
**Age**		-0.003<br>(0.015)	-0.003<br>(0.018)
**Treatment Expectancy**		0.015<br>(0.123)	0.010<br>(0.126)
**Digit Ratio (right)**		-3.319<br>(3.205)	-3.537<br>(3.362)
**Facial Width-Height Ratio**		0.097<br>(0.204)	0.082<br>(0.216)
**Estrone**			0.094<br>(0.196)
**Estradiol**			0.005<br>(0.202)

**DHEA**			-0.335<br>(0.211)
**Cortisol**			-0.090<br>(0.288)
**Cortisone**			0.251<br>(0.400)
**Progesterone**			0.035<br>(0.225)
**Deoxycortisol11**			0.139<br>(0.239)
**Corticosterone**			-0.064<br>(0.414)
**Aldosterone**			0.200<br>(0.272)
**Melatonin**			-0.198<br>(0.304)
**Constant**	4.214***<br>(0.148)	7.090**<br>(3.129)	8.247**<br>(3.629)
**Observations**	242	217	217
**R^2**	0.007	0.017	0.038
**Adjusted R^2**	0.003	-0.016	-0.045
**Residual Std. Error**	2.58 (df=241)	2.60 (df=209)	2.68 (df=199)
**F Statistic**	1.71 (df=1;241)	0.53 (df=7;209)	0.46 (df=17;199)

*p < 0.1 **p < 0.05 ***p <0.01

Regressing the reported die roll on treatment, logged cortisol levels, and the interaction term treatment x logged cortisol did not yield a significant coefficient on the interaction term (beta = 0.217, 95% CI [-0.428, 0.863], $p = 0.508$, R-squared = 0.0095), indicating that cortisol was not a moderator.

Testosterone Level Analysis

Due to nuisance T leading to imprecise measurements in the first 13 of 17 sessions, primary analysis in the manuscript is limited to treatment group. Here we present the results of regressing die rolls on the 4th T sample, which was taken nearest chronologically to the die roll task. M1 is of all sessions, M2 is of the contaminated sessions (1-13), and M3 is of non-contaminated sessions (14-17).

A note on directly comparing our study with Wibral et al. (2012): Wibral et al. (2012) measure serum T levels in blood whereas we use saliva. They used a dose of 50mg of T, whereas our participants received 100mg. Additionally, viral spread of T gel to other people and surfaces is common unless strict measures (akin to ours) are implemented to eliminate the transfer (Kunz et al. 2004; de Ronde 2009). Despite reliable correlations shown between serum and saliva T levels in men of similar age as the participants in our studies (Wang et al., 1982; Morley et al., 2006; Arregger et al., 2006; Lood et al., 2018)), due to substantial T dose differences, and unfortunate unreliability of some of the measures, we do not jointly analyze the relationship between T levels and deception using both studies.

Table S5: Linear Regression using 4th sample, split by session. Dependent variable: die roll. Testosterone levels are log transformed (standard errors in parentheses)

	(M1)	(M2)	(M3)
**TestosteroneD**	-0.0529<br>(0.0448)	-0.0196<br>(0.0524)	-0.169*<br>(0.0991)
**Constant**	4.462***<br>(0.341)	4.197***<br>(0.414)	5.224***<br>(0.651)
**Observations**	241	183	58
**R^2**	0.006	0.001	0.049
**Adjusted R^2**	0.0016	-0.0048	0.0322
**Residual Std. Error**	2.63 (df=241)	2.45 (df=183)	2.44 (df=56)
**F Statistic**	1.39 (df=1;241)	0.14 (df=1;183)	2.89 (df=1;56)

*** p<0.01, ** p<0.05, * p<0.1

The only significant result is a negative relationship between T sample d and deception in the non-contaminated sessions (*p* = 0.09). The coefficients for T sample d (the sample nearest chronologically to the die roll task) and deception in contaminated and contaminated+non-contaminated sessions are negative as well, but not significant.

M4 is of all sessions, M5 is of the contaminated sessions (1-13), and M6 is of non-contaminated sessions (14-17).

Table S6: Linear Regression using all samples, split by session. Dependent variable: die roll. Testosterone levels are log transformed (standard errors in parentheses)

	(M4)	(M5)	(M6)
**TestosteroneA**	-0.165<br>(0.105)	-0.167<br>(0.118)	-0.404<br>(0.414)
**TestosteroneB**	-0.0286<br>(0.0943)	-0.0819<br>(0.111)	-0.0330<br>(0.241)
**TestosteroneC**	-0.170<br>(0.138)	-0.0817<br>(0.157)	-0.439<br>(0.296)
**TestosteroneD**	0.138<br>(0.143)	0.121<br>(0.161)	0.287<br>(0.317)
**Constant**	5.457***<br>(0.672)	5.327***<br>(0.833)	7.361***<br>(2.155)
**Observations**	241	183	58
**R^2**	0.024	0.016	0.107
**Adjusted R^2**	0.0075	-0.0057	0.0391
**Residual Std. Error**	2.57 (df=236)	2.63 (df=178)	2.43 (df=53)
**F Statistic**	1.45(df=4:236)	0.74 (df=4;178)	1.58 (df=4;53)

*** p<0.01, ** p<0.05, * p<0.1

When including all 4 saliva samples, no sample yielded a coefficient statistically significantly different from 0 at the 0.10 level.

8. Joint analysis discussion

Search for Similar Studies

In performing the joint analysis of the results from our study and that of Wibral et al., we first searched for other studies that would be sufficiently similar as to merit inclusion in a full meta-analysis. We chose to limit the scope of what we considered sufficiently similar as to include only studies in which the effects of exogenous T administration in men were measured on a one-shot non-strategic opportunity to deceive in a controlled environment. Our search was also limited to English language articles with human subjects.

To conduct this search, we used both Google Scholar, which as of 2014 was estimated to search through 160 million documents (Orduña-Malea et al., 2015) and Web of Science, which indexes 7 scholarly databases for a total of over 90 million documents as of 2014.

We searched for articles from 1960-2017 using the following combinations of search terms:

1. Dishonesty + testosterone
2. Dishonesty + androgens
3. Lying + testosterone
4. Lying + androgens
5. Cheating + testosterone
6. Cheating + androgens
7. Deception + testosterone
8. Deception + androgens

In addition, we also searched the citations of relevant testosterone studies, such as in Wibral et al. (2012) and van Honk et al. (2016) to find any papers that met our criteria. None did so.

Random Effects Specifications

In the manuscript, we report the result of a fixed effects model. Our result is also robust at the 0.10 level to using a random effects model, d =-0.33 (95% CI[-0.70, 0.05]) and a test of d = 0 is rejected at the 0.10 level (z(1) = 1.71, p = 0.09). The achieved power is > 0.999, calculated using G-Power.

9. References

1. A.L. Arregger et al. Salivary testosterone: a reliable approach to the diagnosis of male hypogonadism. *Clinical endocrinology 67*, 656-662 (2007).R. Horton, J. Tait. Androstenedione production and interconversion rates measured in peripheral blood and studies on the possible site of its conversion to testosterone. *Journal of Clinical Investigation* 45, 301 (1966).
2. J. Y. Du et al., Percutaneous progesterone delivery via cream or gel application in postmenopausal women: a randomized cross-over study of progesterone levels in serum, whole blood, saliva, and capillary blood. *Menopause* 20, 1169 (2013).
3. R.L. Flyckt et al. Comparison of salivary versus serum testosterone levels in postmenopausal women receiving transdermal testosterone supplementation versus placebo. *Menopause 16*, 680-688 (2009).
4. F. Hucklebridge, T. Hussain, P. Evans, A. Clow, The diurnal patterns of the adrenal steroids cortisol and dehydroepiandrosterone (DHEA) in relation to awakening. *Psychoneuroendocrinology* 30, 51 (2005).
5. S. Hurwitz, R. J. Cohen, G. H. Williams, Diurnal variation of aldosterone and plasma renin activity: timing relation to melatonin and cortisol and consistency after prolonged bed rest. *Journal of Applied Physiology* 96, 1406 (2004).
6. G.J Kunz et al., Virilization of young children after topical androgen use by their parents. *Pediatrics* 114.1 282-284 (2004).
7. A.C. Little et al., Symmetry is related to sexual dimorphism in faces: data across culture and species. PloS one 3.5 e2106 (2008).
8. Y. Lood et al. Relationship between testosterone in serum, saliva and urine during treatment with intramuscular testosterone undecanoate in gender dysphoria and male hypogonadism. *Andrology 6*, 86-93 (2018).
9. A. Mayo, H. Macintyre, A. Wallace, S. Ahmed, Transdermal testosterone application: pharmacokinetics and effects on pubertal status, short-term growth, and bone turnover. *The Journal of Clinical Endocrinology & Metabolism* 89, 681 (2004).
10. J.E. Morley et al. Validation of salivary testosterone as a screening test for male hypogonadism. *The Aging Male 9*, 165-169 (2006).
11. G. Nave, A. Nalder, D. Zava, and C. Camerer. Single-dose testosterone administration impairs cognitive reflection in men. *Psychological science 28*, 1398-1407 (2017).
12. S. Nomura, M. Fujitaka, N. Sakura, K. Ueda, Circadian rhythms in plasma cortisone and cortisol and the cortisone/cortisol ratio. *Clinica chimica acta* 266, 83 (1997).
13. E. Orduña-Malea et al. Methods for estimating the size of Google Scholar. *Scientometrics* 104, 931-949 (2015).
14. C. Rolf, U. Knie, G. Lemmnitz, E. Nieschlag, Interpersonal testosterone transfer after topical application of a newly developed testosterone gel preparation. *Clinical endocrinology* 56, 637 (2002).
15. W. De Ronde, Testosterone gel for the treatment of male hypogonadism, *Expert opinion on biological therapy* 9.2 249-253 (2009).
16. J. Van Honk et al. Effects of testosterone administration on strategic gambling in poker play. *Scientific reports* 6, 18096 (2016).

17. C. Wang et al. Salivary testosterone in men: further evidence of a direct correlation with free serum testosterone. *The Journal of Clinical Endocrinology & Metabolism 53*, 1021-1024 (1981).
